# The Adsorption of Chlorpromazine on the Surface of Gold Nanoparticles and Its Effect on the Toxicity to Selected Mammalian Cells

**DOI:** 10.3390/ma17194774

**Published:** 2024-09-28

**Authors:** Magdalena Oćwieja, Anna Barbasz, Oliwia Kowalska, Julia Maciejewska-Prończuk, Agata Lada

**Affiliations:** 1 Jerzy Haber Institute of Catalysis and Surface Chemistry, Polish Academy of Sciences, Niezapominajek 8, PL-30239 Krakow, Poland; oliwia.kowalska@ikifp.edu.pl; 2 Department of Biochemistry and Biophysics, Institute of Biology and Earth Sciences, University of the National Education Commission, Podchorazych 2, PL-30084 Krakow, Poland; anna.barbasz@uken.krakow.pl; 3 Department of Chemical and Process Engineering, Cracow University of Technology, Warszawska 24, PL-31155 Krakow, Poland; julia.maciejewska-pronczuk@pk.edu.pl; 4 Department of Chemistry, Faculty of Mathematics and Natural Sciences, University of Applied Sciences in Tarnow, Mickiewicza 8, PL-33100 Tarnow, Poland; a_lada@atar.edu.pl

**Keywords:** drug–nanoparticle conjugates, gold nanoparticles, neuroleptics, chlorpromazine, toxicity, lymphocytes, melanoma cells, surface charge, surface properties, HUT-78, COLO 720L, COLO 679, B16-F0

## Abstract

Chlorpromazine (CPZ) is a first-generation neuroleptic with well-established antitumor and antiviral properties. Currently, numerous studies are focused on developing new methods for CPZ delivery; however, the knowledge regarding its conjugates with metal nanoparticles remains limited. The aim of this study was to prepare CPZ conjugates with gold nanoparticles (AuNPs) and evaluate their biological activity on human lymphocytes (HUT-78 and COLO 720L), as well as human (COLO 679) and murine (B16-F0) melanoma cells, in comparison to the effects induced by unconjugated CPZ molecules and AuNPs with well-defined properties. During the treatment of cells with CPZ, AuNPs, and CPZ-AuNP conjugates, changes in mitochondrial activity, membrane integrity, and the secretion of lipid peroxidation mediators were studied using standard biological assays such as MTT, LDH, and MDA assays. It was found that positively charged CPZ-AuNP conjugates more effectively reduced cell viability compared to AuNPs alone. The dose-dependent membrane damage was correlated with oxidative stress resulting from exposure to CPZ-AuNP conjugates. The activity of the conjugates depended on their composition and the size of the AuNPs. It was concluded that conjugating CPZ to AuNPs reduced its biological activity, while the cellular response to the treatment varied depending on the specific cell type.

## 1. Introduction

Chlorpromazine (CPZ) is a well-known antipsychotic drug that is widely used in the treatment of diverse mental disorders such as schizophrenia and other types of psychoses (particularly paranoid ones), manic states, and hypomanic states. It also works supportively in the short-term treatment of anxiety states, psychomotor agitation, and violent or dangerous impulsive behaviors [1,2]. From chemical point of view, CPZ is a synthetic phenothiazine (PTZ) derivative containing an additional chlorine atom in one of the benzene rings and a dimethylaminopropyl group on the heterocyclic nitrogen atom [3]. CPZ was synthesized over 70 years ago and undoubtedly revolutionized the treatment of psychotic disorders [4]. It was quickly established that inhibition of dopamine D2 receptors is primarily responsible for the antipsychotic effects of CPZ [1,2]. Despite well-established knowledge about its pharmacokinetic properties, mechanism of action, and side effects, scientific interest in CPZ remains strong. Numerous studies continue to be conducted to gain an even better understanding of its biological activity and still hidden application potential.

One of the areas of interest in CPZ research is its biological activity at the cellular level. It was noticed early on that agranulocytosis and the release of transaminase enzymes from liver cells are highly visible consequences of using neuroleptics such as CPZ, which suggested that synthetic phenothiazine derivatives exert toxic effects on the blood and liver cells [5]. Numerous further research confirmed the cytotoxic activity of CPZ [5,6,7,8,9] and revealed also that it can be a promising anticancer agent [7]. Further research was concentrated on the determination of the mechanisms of the cytotoxic activity of CPZ. For example, Zinetti et al. [8] studied human monocytic leukemia cells and mouse fibrosarcoma L929 cells and discovered that CPZ can influence the secretion of tumor necrosis factor (TNF-α) and apoptosis of the cells. The authors hypothesized that CPZ inhibits the TNF-α pathway in two ways: on the one hand, it inhibits synthesis, and on the other, it inhibits its cytotoxic activity, probably through an interaction with the signaling pathways activated by TNF-α. In turn, the research of Lialiaris et al. [9] revealed that CPZ exhibits an anticancer and cytogenic effect on human lymphocytes in vitro and on Ehrlich ascites tumor (EAT) cells in vivo. The authors found that the combination of CPZ with caffeine and mitomycin C exerted cytostatic and cytotoxic actions in human lymphocytes. Moreover, it was established that the combination of CPZ with mitomycin C exerted cytostatic and cytotoxic actions in EAT cells, significantly increased the survival span of the mice inoculated with EAT cells, and suppressed the expected increase in tumor growth.

Generally, the conducted studies showed that although the antipsychotic mechanism of CPZ is relatively simple, its anticancer action involves a wide range of cellular pathways [10]. It has become clear that, in addition to neuronal receptors, CPZ also affects many seemingly unrelated targets and cellular processes, including, among others, the inhibition of DNA synthesis, uncoupling of oxidative phosphorylation, inhibition of cytochrome oxidase, suppression of adenosine triphosphatase (ATPase) enzymatic activity, alteration of membrane permeability, and inhibition of lipase activity [10]. It is worth mentioning that the continued interest in CPZ is also evidenced by scientific research conducted during the COVID-2019 pandemic, which proved that it has antiviral activity towards SARS-CoV-2 [11]. It was proved that CPZ inhibited the expression and secretion of IL-6 by monocytes activated by SARS-CoV-2 virus nucleocapsid protein and affected the activity of NF-B and MEK/ERK signaling [12]. The researchers proved that CPZ exerted antiviral and immunomodulatory activity and might be a desirable drug to support the therapy of patients with COVID-19 [12].

Numerous reports indicating the anticancer and antiviral activities of CPZ have stimulated researchers to search for new solutions to minimize the side effects of its use in developed anticancer therapies. Analyzing the available literature data, one can observe that during recent years, the works of researchers were oriented towards development of new methods of CPZ encapsulation and delivery. For example, Halayqa and Domańska [13] proposed a new method for encapsulating CPZ in poly(DL-lactide-co-glycolide) (PLGA) nanoparticles. The authors not only developed a new method for delivering CPZ but also demonstrated that this drug can be released from the nanocapsules in a controlled manner [13]. An interesting approach was also proposed by Govender et al. [14] who proposed an encapsulation method for CPZ in poly-ε-caprolactone (PCL)-based nanocapsules. The authors showed that the in vitro CPZ release was biphasic for all formulations with an initial burst of release, followed by pseudo-steady controlled release over 30 days. Moreover, it was proved that the cytotoxicity of the optimized nanocapsule system on a PC12 neuronal cell line proved to be minimal. The conducted research showed that PCL-based nanocapsules filled with CPZ can be applied for the long-term management of certain psychotropic disorders and with this system, the side effects of the neuroleptic were minimalized [14]. In turn, to improve the oral bioavailability of CPZ, Baloch et al. [15] proposed its encapsulation in lipid-based self-nanoemulsifying drug delivery systems (SNEDDSs). The authors showed that the encapsulated CPZ exhibited a 1.5-fold increased elimination half-life (*p* < 0.01), up to a 6-fold increase in oral bioavailability, and a 1.7-fold decrease in its plasma clearance rate (*p* < 0.01) compared to a drug suspension [15].

Another field of research includes conjugations of CPZ with other chemical compounds to extend the range of its application. Panjla et al. [16] showed that the conjugation of CPZ with selected peptides increased the biological activity of the prepared system towards multidrug-resistant *Staphylococcus aureus*. 

The conjugation of CPZ to other colloidal systems seems to be an interesting and promising approach, especially since CPZ is often applied in the form of chloride salts. The CPZ cations can easily bind to other systems, not only via chemical bonds [16] but also through physisorption. It seems plausible that the adsorption of CPZ molecules on the surfaces of colloidal particles can lead to the formulation of new conjugates with combined biological properties of this neuroleptic and nanoparticles (NPs). On one hand, the physisorption of CPZ on the surfaces of hard colloidal particles can facilitate its further release. On the other hand, the positively charged CPZ conjugates can more easily interact with cell membranes. Plasmonic nanoparticles, such as silver, gold, or platinum nanoparticles, seem to be interesting candidates for the preparation of such conjugates. Thanks to the plasmonic properties of metal nanoparticles (MeNPs), it becomes possible to use modern spectroscopic techniques to determine the structure of the drug adsorbed on the metallic surface, even at very low drug concentrations that are undetectable by classical methods [3]. This allows for the determination of the activity of CPZ after the conjugations [3]. The application of surface-enhanced Raman spectroscopy (SERS) and surface-enhanced infrared absorption spectroscopy (SEIRA) allows for the determination of the pH-dependent structure of CPZ conjugated to gold nanoparticles (AuNPs) [3]. Moreover, the stability of such CPZ-AuNP conjugates was assessed based on the SERS and SEIRA spectra. 

The conjugation of drugs to plasmonic nanoparticles opens the door to tracking the fate of drugs in biological fluids and cell organelles, as well as assessing the fate of their metabolites using modern surface-enhanced spectroscopic techniques [17,18,19,20,21]. Nevertheless, the biological activity of conjugates of CPZ with MeNPs has not yet been described in the literature. To fill this gap, the aim of this study was to prepare CPZ-AuNP conjugates with well-defined physicochemical properties and evaluate the toxicity of these systems towards selected mammalian cells. The main hypothesis posits that the adsorption of CPZ molecules onto the surface of negatively charged AuNPs may alter the biological activity of both the drug and the AuNPs, which serve as platforms for the drug’s adsorption. The secondary hypothesis suggests that the toxicity of positively charged CPZ-AuNP conjugates should be comparable to that of other positively charged AuNPs with a similar morphology and size distribution. For the study, two types of human lymphocytes (COLO-720L and HUT-78) were selected, along with human skin melanoma (COLO 679) and murine B16 melanoma (B16-F0) cells.

## 2. Materials and Methods

### 2.1. Chemicals

Hydrogen tetrachloroaurete (III) hydrate, trisodium citrate (TC) dihydrate, sodium borohydride (BH), cysteamine hydrochloride (CH), chlorpromazine (CPZ) hydrochloride, sodium chloride, sodium hydroxide, and hydrochloric acid were obtained from Sigma Aldrich-Merck (Darmstadt, Germany). Poly(allylamine hydrochloride) (PAH, 70 kDa) and natural ruby mica sheets were provided by Polysciences (Niles, IL, USA) and the Continental Trade (Warszawa, Poland), respectively. Quartz/silicon dioxide (Si/SiO_2_) sensors were obtained from the Q-Sense company (Wroclaw, Poland). These chemicals were utilized without further purification. The experiments involved solutions and suspensions prepared using ultrapure water, which was obtained from a Milli-Q Elix & Simplicity 185 purification system (Millipore SA, Molsheim, France). 

### 2.2. Cell Lines and Cell Culture Reagents

B (COLO-720L) and T (HUT-78) lymphocytes were obtained from the American Type Culture Collection (ATCC) (Manassas, VA, USA). The cells were cultured in RPMI 1640 medium containing 10% fetal bovine serum (FBS) and 0.01% penicillin–streptomycin. Murine B16 melanoma cells (B16-F0) and human skin melanoma (COLO 679) cells were supplied by ECACC (Salisbury, UK). B16-F0 cells were grown in DMEM supplemented medium and COLO 679 cells were grown in RPMI 1640 medium (both with 10% fetal bovine serum (FBS) and 0.01% penicillin–streptomycin) at 37 °C in a humidified atmosphere of 5% CO_2_. The culture media, FBS, and antibiotics were purchased from PAN-Biotech GmbH (Aidenbach, Germany).

### 2.3. Synthesis of Gold Nanoparticles (AuNPs)

Two types of citrate-stabilized gold nanoparticles (TC-AuNPs) were prepared according to the Turkevich [22] process and the controlled seed growth method [23]. Smaller TC-AuNPs with an average size of 15 nm (TC-AuNPs15) and larger 55 nm-sized TC-AuNPs (TC-AuNPs55) were prepared according to the protocols described by Bubniene et al. [24] and Gnacek et al. [3], respectively. Cysteamine-stabilized AuNPs with an average size of 15 nm (CHSBAuNPs15) were synthetized based on the methodology described by Oćwieja et al. [25]. Each hydrosol of AuNPs was purified from an unreacted low molar mass compound using an Amicon Stirred Cell 400 (Sigma Aldrich-Merck (Darmstadt, Germany). In the case of TC-AuNPs, the chamber was equipped with a regenerated cellulose membrane (PLHK07610), whereas for CH-AuNPs, a polyethersulfone membrane (Millipore, PBHK07610) was used. The purification process was conducted until the pH of the suspensions attained a value of 5.8. The purification process was also controlled by the measurements of effluent conductivity. The conductivity of the TC-AuNP suspensions and CH-AuNP-15 suspension reached a value of 20 μS/cm and 3 μS/cm, respectively. The pH and conductivity measurements were carried out using a CPC-505 device (Elmetron, Zabrze, Poland) equipped with an ERH-12-6 pH electrode (Elmetron) and EC-60 conductivity sensor (Elmetron).

### 2.4. Preparation of Conjugates of Chlorpromazine (CPZ) with AuNPs

The conjugates of CPZ with AuNPs (CPZ-AuNPs) were prepared using a ligand exchange reaction, following the protocol described previously by Gnacek et al. [3]. Briefly, a 10 mM aqueous solution of CPZ was prepared in Milli-Q water. Then, a controlled volume of AuNPs with a well-defined concentration was added to a given volume of the CPZ solution, which was being mixed on a magnetic stirrer. The mixing was carried out for 20 min at a temperature of 25 °C. By knowing the volumes and concentrations of both solutions, it was possible to produce CPZ-AuNP conjugates with a well-controlled composition [3].

### 2.5. Physicochemical Characteristics of Colloids

The mass concentration of AuNPs in the suspensions and the amount of gold (in the form of unreduced complexes) in the effluents was determined using inductively coupled plasma optical emission spectrometry (ICP-OES). For this purpose, Perkin-Elmer OPTIMA 2100DV equipment (Wellseley, MA, USA) was used. The samples for the measurements were prepared according to the procedure described previously [3].

The spectra of CPZ, the AuNPs, and the conjugates in the UV–vis region were recorded using a UV-2600 spectrometer (Shimadzu, Kyoto, Japan). The morphology and average size of the AuNPs were determined by analyzing micrographs obtained from a JEOL JSM-7500F scanning electron microscope (SEM) equipped with a transmission electron detector (TED). The micrographs were analyzed using MultiScan software v.18.03. A histogram was generated by measuring the surface area and diameter of 1000 AuNPs.

The hydrodynamic diameter (*d*_H_) and electrokinetic properties of the TC-AuNPs and CPZ-AuNP conjugates were determined using a Zetasizer Nano ZS (Malvern Panalytical Ltd., Malvern, UK). The zeta potential values were calculated using Henry’s model based on the electrophoretic mobility (*μ*_e_) measured under controlled pH, ionic strength, and temperature conditions. Each measurement was repeated five times under the specified experimental conditions.

### 2.6. Determination of CPZ Adsorption Process on the Surfaces of AuNPs

The adsorption of CPZ molecules on the surfaces of TC-AuNPs was determined using a gravimetric method. For this purpose, the TC-AuNPs were immobilized on PAH-covered Si/SiO_2_ sensors under flow conditions. The immobilization process was conducted using a Qsense E1 QCM-D system (QSense, Gothenburg, Sweden) according to the procedure described previously [25]. TC-AuNP suspensions with a concentration of 100 mg/L, an ionic strength of 10^−2^ M, and a pH of 5.8 were applied. After the formation of dense TC-AuNP monolayers [25], the system was rinsed with a NaCl solution with an ionic strength of 10^−2^ M and pH of 5.8. In the final step of the investigation, CPZ adsorption on the TC-AuNP monolayer was studied using a CPZ solution with a concentration of 10^−3^ M. The adsorption process was commenced by passing the suspension through the cell at a flow rate of 2.5 × 10^−3^ cm^3^/s. The kinetics of adsorption was measured at a temperature of 25 °C. The experimental results were interpreted based on the Sauerbrey approach described in detail in a previous work [25]. 

For convenience, the methodology for the physicochemical characterization of AuNPs and CPZ-AuNPs is schematically presented in the Supporting Materials (Figure S1).

### 2.7. Cell Viability Assay

Cell viability was determined using the colorimetric 3-(4,5-dimethylthiazol-2-yl)-2,5-diphenyl tetrazolium bromide assay (MTT assay). Cells were seeded in 96-well plates at a density of 0.2 × 10^6^ cells per well in a volume of 0.2 mL/well. After 24 h of exposure to CPZ and AuNPs of a given concentration, 50 μL of an MTT solution (5 mg/L) was added to each well and incubated for 2 h at 37 °C. Subsequently, 0.4 mL of dimethyl sulfoxide (DMSO) was added to the wells. After 5 min, the solutions were centrifuged, and the absorbance of the supernatants at a wavelength of 570 nm was measured using an Epoch microplate reader (BioTek Instruments, Winooski, VT, USA). The cell viability was determined according to a previously described protocol [26].

### 2.8. Membrane Damage Assay

To assess membrane damage, the lactate dehydrogenase (LDH) assay was employed. The cells (at a density of 0.1 million cells per well) were incubated in the presence of CPZ or AuNPs for 24 h. Subsequently, 100 µL of the supernatants were combined with a mixture comprising 10 μL of 0.14 mM NADH and 0.5 mL 0.75 mM sodium pyruvate. Following a 30 min incubation at 37 °C, 0.5 mL of 2,4-dinitrophenylhydrazine was introduced into the solution. After 1 h, the absorbance of the formed hydrazone was measured spectrophotometrically at 450 nm. The absorbance was determined by accounting for the absorbance value from the blank reagent. The release of LDH from cells treated with CPZ and AuNPs was compared to the control group of untreated cells. The data were normalized to the LDH activity released from the control cells, which were vortexed for 5 min and lysed using sonication (5 min, 15 kHz).

### 2.9. Membrane Lipid Peroxidation

The cells (0.2 million per well) treated with CPZ, AuNPs, and CPZ-AuNPs were collected and centrifuged after 24 h of exposure. The cell pellets were homogenized in 5 mL of 0.5% trichloroacetic acid (TCA). After centrifugation, 0.4 mL of the supernatant was added to 1.25 mL of a mixture containing 10% TCA and 0.5% TBA. The mixture was boiled for 30 min in a dry thermoblock and then allowed to cool. The concentration of malondialdehyde (MDA) was determined spectrophotometrically at 532 nm using the molar extinction coefficient of MDA, which is 155 M^−1^ cm^−1^.

### 2.10. Statistical Analysis

The cellular reactions to treatments with CPZ, AuNP, and CPZ-AuNP were examined across five independent replicates for each biochemical assay performed. The resulting data were averaged to calculate the standard deviation. To identify significant differences from the control group, the SAS analysis of variance (ANOVA) procedure was used. Statistical evaluation of the results from each biochemical assay was conducted with the Duncan multirange test, setting the significance level at *p* < 0.05, and was carried out using PC SAS 8.0 software (SAS Institute, Cary, NC, USA). The letters indicate significant (*p* < 0.05) differences between the results from each treatment: TC-AuNPs15—capital letters, TC-AuNPs55—lowercase letters, and CHB-AuNPs15—double lowercase letters

## 3. Results

### 3.1. Physicochemical Characteristics of AuNPs

Three types of AuNP suspensions were obtained using a chemical reduction method with trisodium citrate (TC) and sodium borohydride (SB), both common reducing agents. The mass concentration of gold in the suspensions, as determined by ICP-OES measurements, ranged between 120 and 185 mg/L (Table 1). Additional analyses of the effluents did not reveal the presence of gold, indicating that the suspensions were also free from any low-molecular-mass forms of gold complexes (e.g., unreduced AuCl_4_^−^).

**Figure 1:**
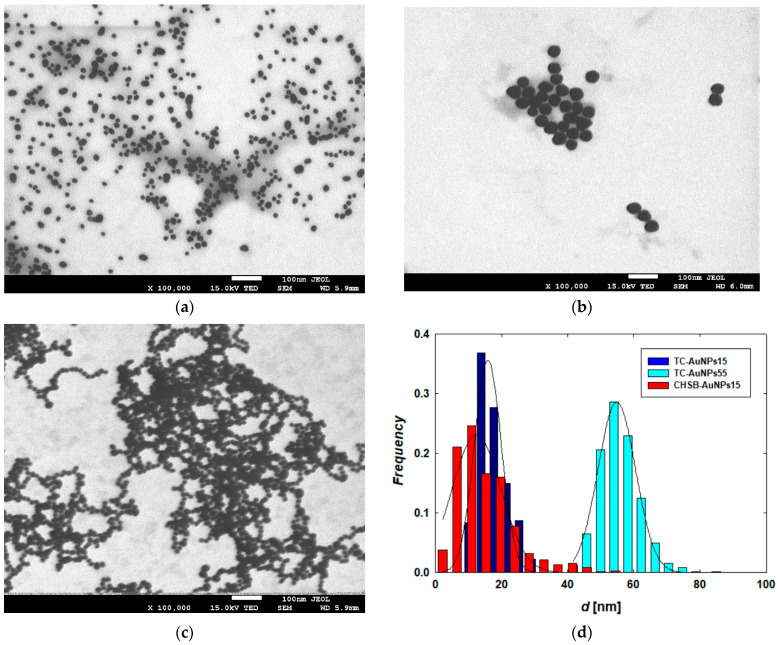
Typical TEM micrographs showing (**a**) TC-AuNPs15, (**b**) TC-AuNPs55, (**c**) CHSB-AuNPs15, and (**d**) size distribution of AuNPs. The histograms were prepared by analyzing the diameters of 1000 AuNPs of each type.

The morphology and size distributions of the AuNPs were determined from the recorded TEM micrographs (Figure 1). It was found that the AuNPs exhibited a quasi-spherical shape and a relatively narrow size distribution (Figure 1d). The average size and polydispersity index (PdI) for each type of AuNP are presented in Table 1. The physicochemical characterization of the AuNPs also included the determination of their hydrodynamic diameter (*d*_H_) and electrokinetic properties (electrophoretic mobility and zeta potential) under controlled pH, ionic strength, and temperature conditions. Considering that the obtained results were consistent with previous literature data [3,24,25,27,28], this study focused on the properties of the AuNPs at a pH of 7.4 and a temperature of 37 °C, as these conditions are typical for biological studies. The results are collected in Table 1. Notably, two types of negatively charged citrate-stabilized AuNPs with average sizes of 17 ± 5 nm and 55 ± 5 nm, as well as one type of positively charged cysteamine-capped AuNPs with an average size of 15 ± 6 nm, were used for further investigation.

**Figure 2:**
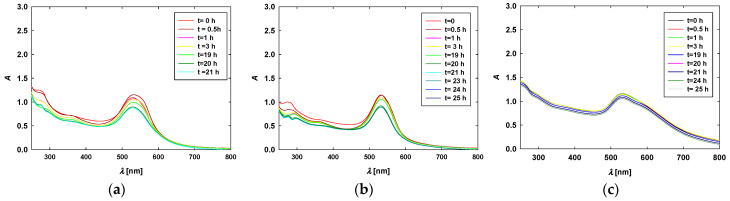
Time-dependent extinction spectra of (**a**) TC-AuNPs15, (**b**) TC-AuNPs55, and (**c**) CHSB-AuNPs15 dispersed in 10% FBS-supplemented culture medium. The concentration of the AuNPs was equal to 50 mg/L; the storage temperature was 37 °C.

Taking into account the importance of AuNP stability in culture media, additional studies included time-dependent measurements of the extinction spectra of AuNPs dispersed in the full culture media were performed. The results of these studies are shown in Figure 2.

Analyzing the recorded extinction spectra, it can be observed that their main features remained unchanged over time. For each type of AuNP, the position and intensity of the maximum absorption bands were relatively stable and comparable to those recorded for AuNPs dispersed in pure aqueous solutions (without the addition of low- and high-molecular-mass compounds). Based on these results, it can be concluded that the AuNPs dispersed in the 10% FBS-supplemented culture medium did not aggregate.

### 3.2. Physicochemical Characteristics of CPZ-AuNP Conjugates

**Figure 3:**
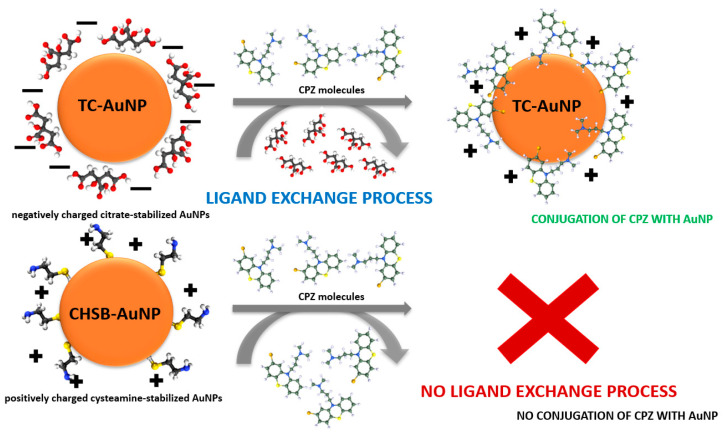
Schematic illustration of a ligand exchange process on the surface of a AuNP.

The preparation of CPZ-AuNP conjugates was based on the ligand exchange process [3] schematically presented in Figure 3. For negatively charged TC-AuNPs15 and TC-AuNPs55, citrate anions are physisorbed onto the gold surfaces [29]. In the presence of CPZ molecules, which are delivered as chlorpromazine hydrochloride, these citrate anions are displaced from the surface, allowing the adsorption of CPZ cations, which leads to the formation of CPZ-AuNP conjugates [3]. In this case, the ligand exchange process is electrostatically driven due to the cationic form of CPZ. Conversely, the use of positively charged CHSB-AuNPs15 does not lead to the formation of CPZ-AuNP conjugates. On one hand, the formation of CPZ-AuNPs is inhibited by repulsive electrostatic interactions between CPZ cations and protonated cysteamine (CH) molecules adsorbed on the CHSB-AuNP surfaces. On the other hand, cysteamine molecules are chemisorbed [25] on the surface of CHSB-AuNPs, preventing the ligand exchange process from occurring.

In a previous literature report, the electrokinetic and spectroscopic properties of CPZ-AuNP conjugates formed using TC-AuNPs55 were described in detail [3]. It was shown that CPZ adsorption on TC-AuNPs55 occurs through the angularly oriented phenothiazine moiety and the CN-C bond, which is vertically oriented relative to the AuNP surface. Additionally, it was established that the electrokinetic properties of CPZ-AuNP conjugates depend on the mass ratio of the components as well as the pH [3]. These relationships are independent of the size of the AuNPs; therefore, the electrostatically driven preparation of two types of positively charged conjugates, designated as CPZ-AuNPs15 and CPZ-AuNPs55, was easily accomplished.

The selected physicochemical properties of these conjugates are collected in Table 2. Additionally, the adsorption process of CPZ molecules on the surface of TC-AuNPs15 and TC-AuNPs55 immobilized on PAH-covered QCM sensors was studied using (Supporting Materials, Figure S2). For this purpose, negatively charged TC-AuNPs were adsorbed onto PAH-modified Si/SiO_2_ sensors at an ionic strength of 10^−2^ M NaCl, which allowed for the formation of dense and homogeneous TC-AuNP monolayers. The coverage of TC-AuNPs15 and TC-AuNPs55, expressed as the mass of adsorbed nanoparticles per unit area, was 115 and 205 mg/m^2^, respectively (Supporting Materials, Figure S2). These values correspond to a dimensionless coverage of 0.32–0.33 [25,27]. After forming the TC-AuNP monolayers, their surfaces were activated by flowing a 10^−2^ M NaCl solution over them [30]. Subsequently, CPZ molecules were deposited onto the monolayers using a 10^−3^ M CPZ solution (Supporting Materials, Figures S2 and S3). The deposition of CPZ molecules, conducted at a flow rate of 1.33 × 10^−3^ cm^3^/s, lasted for 30 min. After this period, the stability of the CPZ layers deposited on the AuNPs was assessed by passing a 10^−3^ M sodium chloride solution over the prepared systems. The rinsing procedure continued for an additional 30 min, and no desorption of CPZ molecules was observed. Thus, the stability of the CPZ-AuNP system was confirmed not only through spectroscopic studies [3] but also via QCM measurements.

**Figure 4:**
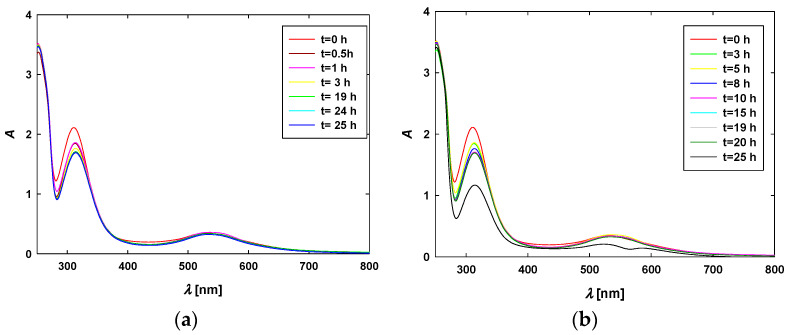
Time-dependent extinction spectra of (**a**) CPZ-AuNPs15 and (**b**) CPZ-AuNPs55 dispersed in 10% FBS-supplemented culture medium. The concentration of the TC-AuNPs was equal to 50 mg/L and the concentration of CPZ was 10^−3^ M; the storage temperature was 37 °C.

In the next stage of the studies, the stability of CPZ-AuNP conjugates in culture media was assessed in a manner similar to the experiments conducted with the AuNPs. The results are presented in Figure 4. Analyzing the spectra, one can observe two characteristic bands at wavelengths of 250 and 314 nm originating from CPZ, along with bands from the TC-AuNPs located at 532–534 nm. These bands are slightly shifted to higher wavelengths compared to the bands detected for the pure components of the conjugates dispersed in aqueous medium. This effect arises from the presence of various components in the culture medium, which alter the dielectric constant of the medium surrounding the AuNPs. Nevertheless, no significant differences in the position or intensity of these bands were detected. This confirms the stability of the CPZ-AuNP conjugates dispersed in the culture medium.

### 3.3. Cytotoxicity of CPZ and AuNPs towards Human Lymphocytes and Melanoma Cells

The main aim of the study was to determine the toxicity of CPZ and its conjugates with the AuNPs towards selected mammalian cells. The attention was focused on human T lymphocytes (HUT-78) and B lymphocytes (COLO 720L), which are extremely important cells of the immune system that are found in the blood and lymphatic tissues. Lymphocytes are responsible for recognizing antigens (viruses, bacteria, and other pathogens) and reacting to destroy them. Additionally, it was observed early in CPZ’s use as a drug that it significantly affects the function of these essential cells [9,31,32,33,34]. The second group of cells studied comprised melanoma tumor cells, specifically murine B16 melanoma cells (B16-F0) and human skin melanoma cells (COLO 679). Similarly to lymphocytes, the effects of CPZ on various types of melanoma cells have been investigated [10,35,36,37]. However, the biological activity of CPZ conjugated with AuNPs has not yet been described or compared to the effects induced by free CPZ molecules. The results of the biological study here can help clarify this issue.

In the first stage of the studies, the toxicity of CPZ was evaluated by investigating changes in mitochondrial activity using an MTT assay. The results, which show the dependence of cell viability on CPZ dose for lymphocytes and melanoma cells, are presented in Figure S4 (Supporting Materials). As expected, a reduction in cell viability due to 24 h exposure to CPZ occurred in a dose-dependent manner. For lymphocytes, treatment with a 10^−3^ mM CPZ solution reduced the viability of HUT-78 and COLO 720L cells to 88% and 82%, respectively. Increasing the CPZ concentration by three orders of magnitude to 1 mM resulted in a further reduction in cell viability to 21% and 18%, respectively (Figure S4a). A similar CPZ-dose-dependent reduction in cell viability was observed in melanoma cells, although these cells appeared to be less sensitive to the CPZ treatment than the lymphocytes. For COLO 679 cells, a 24 h exposure to 10^−3^ mM CPZ reduced their viability to 83%, while a concentration of 1 mM led to a decrease in viability to 36% (Figure S4b). Murine B16 melanoma cells proved to be the most resistant to CPZ, particularly at higher concentrations. For example, at CPZ concentrations of 1 mM and 10 mM, the viability of B16-F0 cells remained at 57% and 46%, respectively. Since the aim of these studies did not include determining the mechanisms of CPZ activity, which have been detailed in other literature reports [10], the subsequent research focused on evaluating the biological activity of AuNPs with diverse physicochemical properties (Table 1). This was followed by an investigation of the AuNPs’ activity after CPZ adsorption onto their surface and the formation of CPZ-AuNP conjugates. 

**Figure 5:**
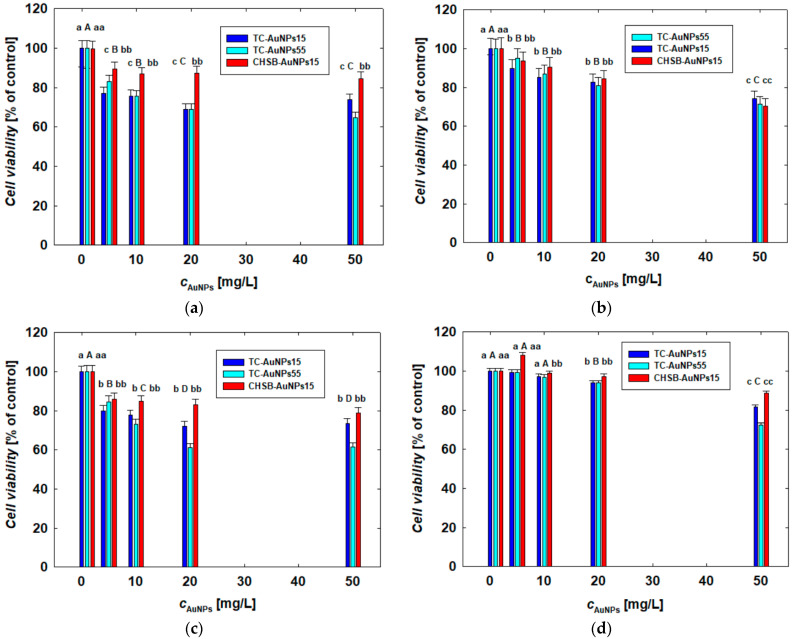
Effect of AuNPs on viability of (**a**) HUT-78 and (**b**) COLO 720L lymphocytes and (**c**) COLO 679 and (**d**) B16-F0 melanoma cells. The viability of the cell lines was determined after 24 h of the AuNP treatment using the MTT assay. The letters indicate significant (*p* < 0.05) differences between the results from each treatment.

The response of the cells to different AuNP concentrations was also investigated after 24 h using the MTT assay. The results, for each type of cell, are shown in Figure 5. The AuNP concentrations applied in this research ranged from 1 to 50 mg/L. Within this range, the cell viability did not change significantly. The lowest cell viability was observed in COLO 679 cells after a 24 h treatment with TC-AuNPs55 at a concentration of 50 mg/L, where the cell viability dropped to 61% (Figure 5c). A key finding from this research is that the cell response to the AuNP treatment depended on the physicochemical properties of the AuNPs. Notably, positively charged CHSB-AuNPs15 were generally less toxic to the cells compared to negatively charged citrate-stabilized TC-AuNPs of both sizes. At a concentration of 50 mg/L, the cell viability remained at 89% and 71% for B16-F0 and COLO 720L cells, respectively. Significant differences in toxicity between TC-AuNPs15 and TC-AuNPs55 were not observed up to a concentration of 20 mg/L. However, at the highest investigated concentration, the larger TC-AuNPs55 were more effective in reducing cell viability compared to TC-AuNPs15. This trend was not observed for COLO 720L cells, where the effects induced by each type of AuNP were similar regardless of the concentration (Figure 5b).

### 3.4. Cytotoxicity of CPZ-AuNP Conjugates towards Human Lymphocytes and Melanoma Cells

**Figure 6:**
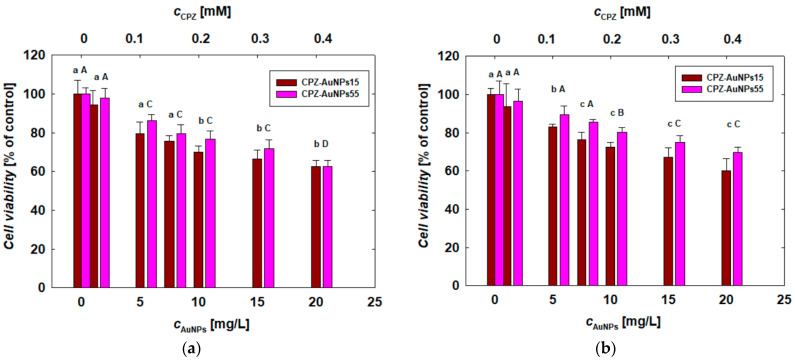

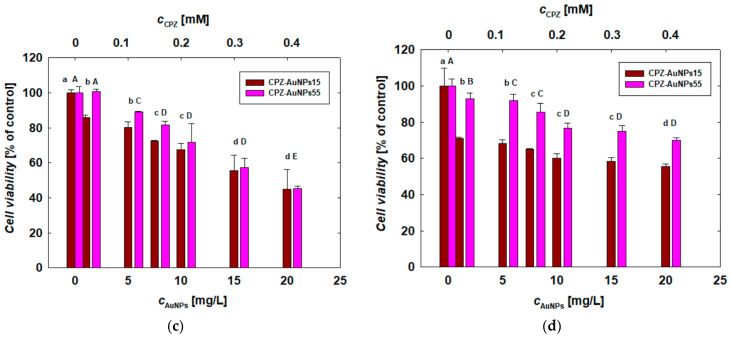
Effect of CPZ-AuNP conjugates on viability of (**a**) HUT-78 and (**b**) COLO 720L lymphocytes and (**c**) COLO 679 and (**d**) B16-F0 melanoma cells. The viability of the cell lines was determined after 24 h of the CPZ-AuNP treatment using the MTT assay. The letters indicate significant (*p* < 0.05) differences between the results from each treatment.

Both suspensions of the CPZ-AuNP conjugates were prepared in the same manner, with the Au concentration at 50 mg/L and the CPZ concentration at 1 mM (356 mg/L) (Table 2). Given that the conjugates consist of two biologically active substances, the results of the toxicity studies were interpreted by comparing the effects induced by the CPZ-AuNPs with those caused by the AuNPs and free CPZ. Additionally, attention was focused on ensuring that the concentrations of both components were consistent across the pure AuNP suspensions, CPZ solutions, and CPZ-AuNP suspensions. At the beginning, the cells were exposed to both types of CPZ-AuNP conjugates. After 24 h of treatment, cell viability was determined based on changes in mitochondrial activity, as measured by the MTT assay. The results of the investigation are presented in Figure 6. The first noticeable observation is that the response of the cells to the CPZ-AuNP conjugate treatment was dose-dependent—similar to the case of free CPZ molecules and AuNPs (Figure S4, Supporting Materials, and Figure 5).

Generally, a reduction in the viability of all cell types was observed with an increase in the concentration of the conjugates. Another noticeable trend was the generally lower toxicity of CPZ-AuNPs55 compared to CPZ-AuNPs15. For instance, exposure of COLO 720L cells to CPZ-AuNPs55 at the highest concentration resulted in a viability decrease to 68%, whereas with CPZ-AuNPs15, the viability dropped to 58%. A similar trend was observed in B16-F0 cells treated with the highest concentration of the CPZ-AuNP conjugates, where the cell viability was reduced to 70% and 59% for CPZ-AuNPs55 and CPZ-AuNPs15, respectively. In the case of HUT-78 and COLO 679 cells, the lower toxicity of the CPZ-AuNPs was less apparent at the highest conjugate concentration but was more evident at lower doses of the conjugates (Figure 6).

Considering the cell sensitivity to the action of CPZ-AuNP conjugates, one can observe that both types of lymphocytes exhibited a comparable response. The cell viability remained between 58 and 70% after treatment with the highest dose (Figure 6a,b). Both types of conjugates caused the greatest decrease in viability in the human melanoma cells (COLO 679), while the murine melanoma cells were the more resistant to the CPZ-AuNP treatment among all the tested cells.

In the next stage, the comparison focused on the toxicity effects induced by the AuNPs (Figure 5) and CPZ-AuNP conjugates (Figure 6), as determined by the results of the MTT assay. When comparing two systems with the same size distribution of the metallic core—specifically, TC-AuNPs15 and CPZ-AuNPs15—it can be observed that the adsorption of CPZ molecules on the surface of citrate-stabilized AuNPs led to an enhanced cytotoxic effect in comparison to AuNPs.

For a given AuNP mass concentration, considering both TC-AuNPs15 and CPZ-AuNPs15, the conjugates caused a greater reduction in cell viability than the AuNPs. For example, at an AuNP concentration of 20 mg/L, the viability of HUT-78 cells was 72% for TC-AuNPs15 and 62% for CPZ-AuNPs15. A similar trend was observed in melanoma cells, with a more pronounced effect in COLO 679 cells. At the same concentration of 20 mg/L, the viability of human melanoma cells decreased to 73% with TC-AuNPs15 and 46% with CPZ-AuNPs15. Overall, for a given gold concentration, the reduction in cell viability was 10 to 37% greater after treatment with CPZ-AuNPs15 compared to TC-AuNPs15.

The trend observed for the pair of TC-AuNPs15 and CPZ-AuNPs15 was also maintained in the pair of TC-AuNPs55 and CPZ-AuNPs55. Despite the fact that the size of both citrate-stabilized AuNPs and CPZ-AuNP conjugates was the same, at approximately 55 ± 5 nm, the positively charged conjugates reduced cell viability more effectively than the negatively charged AuNPs. At an AuNP concentration of 20 mg/L, the viability of HUT-78 cells treated with TC-AuNPs55 and CPZ-AuNPs55 was 68% and 61%, respectively, whereas for B16-F0 cells, it was 93% and 69%, respectively.

Based on these findings, it can be deduced that the adsorption of CPZ molecules on the surface of AuNPs leads to the formation of CPZ-AuNP conjugates with a greater toxic effect than that observed for unmodified AuNPs. Initially, this enhancement in cytotoxicity can be attributed to the acquisition of a positive charge by the AuNPs due to the adsorption of CPZ cations on their surface [3]. For this reason, a comparison was made between the effects induced by positively charged CHSB-AuNPs15 and CPZ-AuNPs15, which have a comparable size distribution of the metallic core and similar electrokinetic properties (Table 1 and Table 2), but differ in their stabilization chemistry (different stabilizers) and stabilization method (CH chemisorption vs. CPZ physisorption). The results obtained for the highest AuNP concentration of 20 mg/L revealed that, for each type of tested cell, the CPZ-AuNPs15 conjugates were more toxic than CHSB-AuNPs15.

It was found that melanoma cells were more sensitive to the effects of CPZ-AuNPs15 than CHSB-AuNPs15. For COLO 679 cells, the viability after 24 h of treatment was 83% for CHSB-AuNPs15 and 44% for CPZ-AuNPs15. In contrast, the differences induced by both types of positively charged systems were noticeably smaller in human lymphocytes. For COLO 720L cells, the viability remained at 84% for CHSB-AuNPs15 and 68% for CPZ-AuNPs15. These findings indicate that the toxicity of both investigated systems strongly depends on the type of stabilizing agents adsorbed on the surface of the AuNPs. However, when comparing these results, it is important to consider both the concentration and toxicity of the compounds used, namely cysteamine (CH) and CPZ. To discuss these parameters, we first need to consider the composition of the CPZ-AuNPs15 conjugates. The highest dose of conjugates used in the biological tests corresponded to AuNPs and CPZ concentrations of 20 mg/L and 0.4 mM, respectively (Figure 4). A two-fold dilution resulted in a conjugate suspension with AuNPs and CPZ concentrations of 10 mg/L and 0.2 mM, respectively. Considering this issue, it is useful to re-consider the differences in toxicity between CHSB-AuNPs15 and CPZ-AuNPs15 at the same AuNP concentration. At an AuNP concentration of 20 mg/L, the differences in toxicity between the two systems ranged from 16% to 39%, whereas at 10 mg/L, the differences ranged from 9% to 12%. Thus, as the AuNP concentration increased, the differences in toxicity between CHSB-AuNPs15 and CPZ-AuNPs15 became more pronounced. For CHSB-AuNPs15, the dose-dependent reduction in cell viability observed across the concentration range was not highly significant, varying between 98% and 73% for concentrations ranging from 1 to 50 mg/L. In contrast, for CPZ-AuNPs15 conjugates, the cell viability ranged from 92% to 45% for AuNP concentrations, it ranged from 1 to 20 mg/L. This indicates that within this narrower AuNP concentration range, the changes in cell viability caused by the CPZ-AuNPs15 conjugate were significantly greater compared to CHSB-AuNPs15, despite both having similar sizes and electrokinetic properties. These results suggest that in the CPZ-AuNPs15 system, the concentration of CPZ also plays a crucial role. In the biological studies, the CPZ concentration ranged from 0 (Figure 5) to 0.4 mM (Figure 6) for both types of CPZ-AuNP conjugates. The CPZ concentration in the conjugate suspension is indicated on the upper axis of Figure 6.

Since the viability of the selected mammalian cells after CPZ treatment was previously measured using the MTT assay (Supporting Materials, Figure S4), it was possible to compare the effects of CPZ at a given concentration, both as free molecules and when conjugated to AuNPs of two different sizes. After 24 h of treatment, the lymphocyte viability with CPZ at a concentration of 1 mM fluctuated between 18% and 22%, while melanoma cell viability ranged from 26% to 57%. Reducing the CPZ concentration by one order of magnitude to 0.1 mM did not significantly change the lymphocyte viability, which remained at 24% to 26%. However, the effect was noticeable in the case of melanoma cells. The viability of human melanoma cells (COLO 679) after 24 h of treatment with CPZ at a concentration of 0.1 mM remained at 37%. For murine melanoma cells (B16-F0), the viability under these conditions was higher, at 62%. With these results in mind, one can compare the viability of each cell type with the values observed for CPZ conjugated to AuNPs of different sizes. For CPZ-AuNPs15 suspensions containing 0.1 mM CPZ, the lymphocyte viability ranged from 78% to 83%, while for melanoma cells, it ranged from 68% to 79%.

For CPZ-AuNPs55, which have a larger gold core but the same gold and CPZ content as CPZ-AuNPs15, it was observed that the viability of each cell type reached 89%. Based on these results, it can be concluded that the conjugation of CPZ molecules to AuNPs reduces its toxic effect. For a CPZ concentration of 0.1 mM, this relationship is significant for human cells, while it is less pronounced for murine melanoma cells when CPZ is conjugated with TC-AuNPs15. Given that the cell viability for CPZ-AuNPs with 0.1 mM CPZ remained at 80%, and considering the effect of AuNPs at a concentration of 5 mg/L and comparing this with the viability found after treatment with pure CPZ (Supporting Materials, Figure S4), it can be concluded that conjugation reduces CPZ toxicity by approximately tenfold.

**Figure 7:**
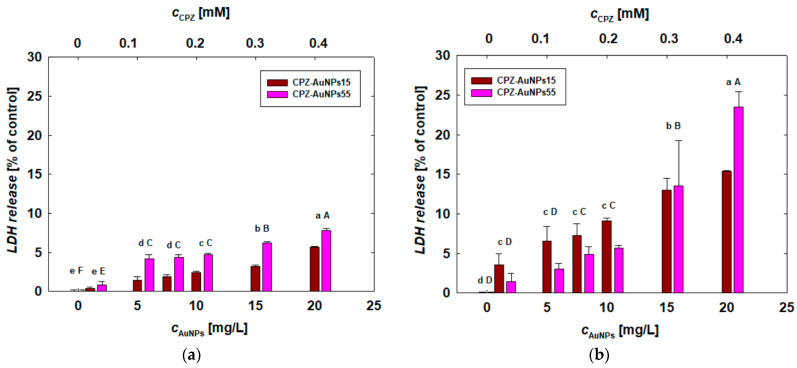

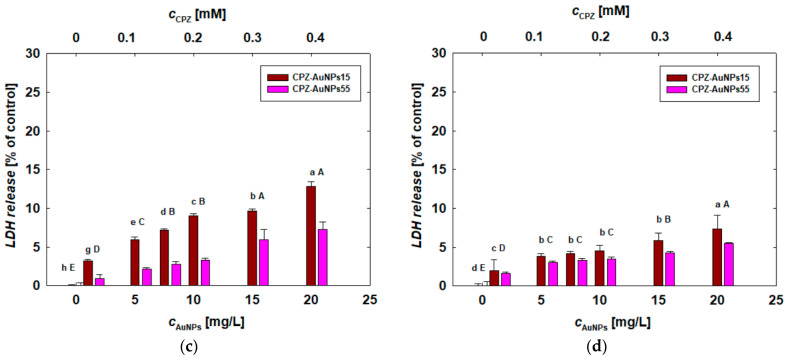
Effect of CPZ-AuNP conjugates on the secretion of LDH by (**a**) HUT-78 and (**b**) COLO 720L lymphocytes and (**c**) COLO 679 and (**d**) B16-F0 melanoma cells. The disruption of the cell membranes was determined after 24 h of the CPZ-AuNP treatment using the LDH assay. The letters indicate significant (*p* < 0.05) differences between the results from each treatment.

The biological activity of the CPZ-AuNPs was also evaluated based on their effect on cell membrane integrity. To determine this, the LDH assay was employed to measure the impact of positively charged conjugates on membrane disruption in the cells. The results of this investigation are shown in Figure 7. In the absence of exposure to CPZ-AuNP conjugates, no LDH release was observed. However, even at the lowest dose of the conjugates tested, the release of this cell damage marker was strongly detectable. At the lowest dose, the amount of LDH released ranged from 1.74% to 3.4% of the control in HUT-78 and COLO 720L cells, respectively. The secretion of LDH increased significantly with the conjugate dose, and this trend was consistent across all cell types. From analyzing the study results, it was evident that the membranes of COLO 720L cells were the most sensitive to exposure to both types of conjugates. For these lymphocytes, the LDH release was the highest, particularly at the highest dose tested. In the case of the CPZ-AuNPs15 and CPZ-AuNPs55 conjugates, the LDH release reached 15% and 24%, respectively (Figure 7b).

It is difficult to identify a clear dependency of conjugate size on LDH secretion, which reflects cell damage. The CPZ-AuNPs55 conjugates caused more significant cell membrane damage only in HUT-78 cells. For both melanoma cell types, the smaller CPZ-AuNPs15 conjugates led to greater membrane disintegration and higher LDH secretion. In the case of COLO 720L cells, at lower doses, the effect induced by CPZ-AuNPs15 was more pronounced, whereas at the highest dose, CPZ-AuNPs55 resulted in greater LDH secretion (Figure 7b).

Given that the loss of cell membrane integrity could result from membrane lipid peroxidation caused by oxidative stress, the next phase of the research involved utilizing the MDA assay to assess this process. It was found that the conjugates induced lipid peroxidation in each type of investigated cell.

**Figure 8:**
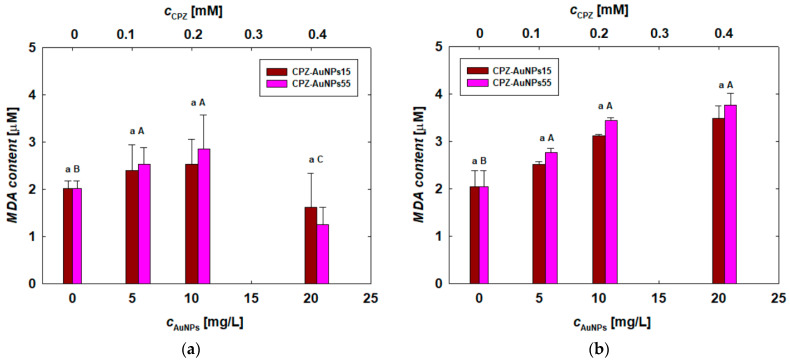

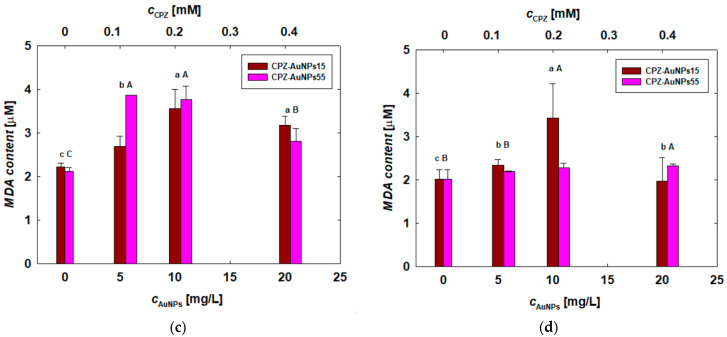
The extent of peroxidation of membrane lipids, expressed by MDA content, in (**a**) HUT-78 and (**b**) COLO 720L lymphocytes and (**c**) COLO 679 and (**d**) B16-F0 melanoma cells. The letters indicate significant (*p* < 0.05) differences between the results from each treatment.

The highest MDA concentrations were detected in COLO 720L and COLO 679 cells. Interestingly, in the case of B lymphocytes, the MDA content increased significantly with the increase in conjugate concentration (Figure 8b), whereas this trend was not observed in human melanoma cells (Figure 8c). It was also notable that the elevated MDA secretion in these cells closely corresponded to the release of LDH (Figure 7b,c). Therefore, it can be inferred that the disruption of the cell membrane due to exposure to the CPZ-AuNP conjugates was correlated with the oxidative stress occurring in the cells, as indicated by the increased MDA secretion.

## 4. Discussion

Despite the wide range of successful applications of CPZ in the treatment of various mental disorders, cancer, and viral diseases, it has been established that this drug induces many side effects, such as irregular heartbeat, blood pressure problems, fever, and rash. To reduce these inconveniences and improve its biological activity depending on the medical destination, numerous approaches to CPZ delivery have been developed. Recently, scientific attention has strongly focused on the encapsulation of CPZ in various polymeric nano- and microcapsules [13,14,15,16]. Numerous studies have demonstrated the successful encapsulation of CPZ inside colloidal particles and its controlled release under specific pH, ionic strength, or temperature conditions [13,14,15,16]. To the best of our knowledge, the formulation of CPZ immobilized on the surface of colloidal particles has not yet been described. Therefore, our research focused on developing the preparation of CPZ conjugates with AuNPs and evaluating their biological activity.

For several reasons, AuNPs are especially promising for conjugation with CPZ. First of all, AuNPs, like CPZ, are capable of crossing the blood–brain barrier [38,39]. Moreover, they exhibit localized surface plasmon resonance (LSPR), which allows them to detect various analytes at very low concentrations [40]. Therefore, conjugating CPZ with AuNPs makes it possible to track the drug’s distribution in different organs and at the cellular level using advanced spectroscopic techniques such as SERS, SEIRA, or TERS. Unlike other plasmonic particles, such as silver or copper NPs, AuNPs are more inert and less biologically active [41]. Additionally, AuNPs can act as contrast agents, highlighting specific organ regions where they accumulate—even when conjugated with drugs.

It has been established that some neuroleptics from the phenothiazine group can induce neurodegenerative diseases, such as drug-induced parkinsonism [42]. In contrast, some AuNPs can influence neurodegenerative diseases by inhibiting protein fibrillation processes [43]. Therefore, it seems plausible that conjugates of CPZ with AuNPs could be promising candidates for reducing the side effects associated with free CPZ molecules. For these reasons, the conjugation of CPZ with AuNPs appears to have significant application potential in the treatment of neurodegenerative diseases.

Herein, two types of citrate-stabilized AuNPs have been selected for the conjugation of CPZ. Trisodium citrate is one of the most common low-molecular-weight stabilizers of plasmonic nanoparticles, including silver, gold, and platinum nanoparticles. Negatively charged metal nanoparticles stabilized by citrate anions are well-documented in the literature and are widely available [44]. Many scientific studies have focused on metal nanoparticles stabilized with citrate anions, and they are also commercially accessible. As a result, they are often considered a model colloidal system for biological studies. Based on this knowledge, two types of spherical AuNPs with an average size 15 nm and 55 nm were prepared for the studies. This selection was made to be able to compare the effects of CPZ conjugates formed on AuNPs of two different sizes. This approach allowed for the evaluation of the effect of the size of the conjugates on their biological activity.

It is worth mentioning that the conjugates of CPZ with AuNPs, due to the low molar mass of CPZ can be considered as AuNPs stabilized by CPZ molecules [3]. Therefore, in order to better understand the role of the stabilizing layer on the biological activity of AuNPs, for this study, cysteamine-stabilized AuNPs were selected. Cysteamine (CH) was selected due to its excellent chemisorption ability on gold surfaces, as demonstrated in our previous work and supported by other literature reports [25,28]. Additionally, using cysteamine, one can produce positively charged AuNPs. Due to the surface charge of the particles, CHSB-AuNPs15 were similar to CPZ-AuNP conjugates. It is worth mentioning that the positive surface charge of CHSB-AuNPs15 and CPZ-AuNPs resulted from the protonation of the amino groups in the CH and CPZ molecules. Therefore, we chose CH as a stabilizer due to its similar protonation potential to CPZ. 

The collection of two types of oppositely charged AuNPs with similar size distributions was important for determining the role of surface charge in modulating cytotoxic effects. Comparing the effects induced by CHSB-AuNPs and CPZ-AuNPs, both with the same surface charge and size distribution, helps describe the impact of stabilizers on the observed toxicity towards selected cells.

In selecting cell lines, we were guided by the literature where many investigations into the biological activity of CPZ have been conducted using lymphocytes and melanoma cells [31,32,33,34,35,36,37]. For this reason, these cells were chosen as reference systems to compare the effects described in the literature for free CPZ molecules with those caused by its conjugates. It is also worth noting that lymphocytes are crucial components of the human body’s defense system. They are widely distributed in the blood and tissues and frequently encounter ‘foreign’ agents, including AuNPs. The body’s response to toxic agents often depends on these cell types, as they are among the first to interact with foreign substances. Therefore, selecting lymphocytes is crucial from a practical standpoint and for further application of CPZ-AuNP conjugates.

The preparation conditions of the colloidal particles were selected to obtain two types of negatively charged citrate AuNPs, with average sizes of 17 ± 5 nm and 55 ± 5 nm, and one type of positively charged AuNPs with an average size of 15 ± 6 nm (Table 2). This resulted in two pairs of AuNPs: the first pair with diverse sizes but identical surface properties, and the second pair with comparable sizes but diverse surface properties. The citrate-stabilized AuNPs were used to obtain two types of CPZ-AuNP conjugates characterized by different sizes but identical electrokinetic properties (Table 1). The insightful physicochemical characterization revealed that the CPZ-AuNP conjugates were stable. Based on the recorded UV–vis spectra, it was established that the AuNPs and conjugates do not aggregate when dispersed in the culture medium (Figure 4). Moreover, investigations using a QCM showed that the desorption of CPZ from the surfaces of AuNPs did not occur (Figure S3), confirming that the conjugates were stable.

With well-defined and stable AuNPs and CPZ-AuNP conjugates, biological studies were conducted. Firstly, the impact of CPZ on the cells was determined (Figure S4). It was found that CPZ reduced the viability of each cell type in a dose-dependent manner. These results were consistent with previous literature reports [6,34], which showed the adverse effects of this drug when applied at higher doses [5] and in the presence of UV radiation [45].

The prepared AuNPs, considered as substrates for the CPZ conjugates, exhibited toxicity that was strongly dependent on their physicochemical properties (Table 1, Figure 5). It was established that positively charged cysteamine-stabilized AuNPs (CHSB-AuNPs15) were significantly less inert to the cells than both types of negatively charged AuNPs. Although cysteamine is a highly toxic substance, its chemisorption on the surfaces of AuNPs inhibited the activity of the thiol moiety, thereby reducing its toxicity. A similar effect was previously observed in comparative studies conducted on cysteamine- and citrate-stabilized silver nanoparticles (AgNPs) with comparable size distributions. Positively charged AgNPs were less toxic to leukemia cells than negatively charged ones [46].

Considering the size effect of citrate-stabilized AuNPs, no significant influence was found on the mitochondrial activity of the tested cells (Figure 5). However, the size effect was clearly noticeable in the treatments conducted with both types of CPZ-AuNP conjugates. In general, smaller CPZ-AuNP conjugates decreased cell viability more significantly than larger ones (Figure 6). Moreover, they were more toxic than CHSB-AuNPs, which also exhibited a positive surface charge. The CPZ-AuNP conjugates affected mitochondrial activity and induced oxidative stress, leading to the peroxidation of membrane lipids and, consequently, disintegration of the cell membrane. The conjugate-dependent LDH leakage varied depending on the cell type. COLO 679 cells were the most sensitive to the action of the conjugates, as detected by the LDH release (Figure 7). The conjugates induced oxidative stress in the cells, which caused damage to the cell membrane through lipid peroxidation.

Based on the obtained results, it was established that the biological activity of the conjugates strongly depends on the size of the gold core. The immobilization of CPZ molecules on the surface of TC-AuNPs increases toxic effects regardless of nanoparticle size. Considering the differences between the effects induced by AuNPs with the same surface charge but different chemical structures of stabilizing layers, it can be concluded that the biological activity and the nature of their immobilization on the nanoparticle surface are pivotal factors influencing the toxicity.

## 5. Conclusions

Two types of CPZ conjugates with AuNPs, characterized by average sizes of 17 ± 5 nm and 55 ± 5 nm, were prepared using an electrostatically driven ligand exchange process. As a result of this method, the composition of the CPZ-AuNP conjugate suspensions was well-defined. The physicochemical properties of the CPZ-AuNP conjugates were analyzed using TEM imaging, DLS, ELS, and UV–vis spectrometry. It was found that both types of conjugates, designated as CPZ-AuNPs15 and CPZ-AuNPs55 based on the size of their gold cores, were positively charged and remained stable for 24 h during storage in the culture media used for the biological tests. In contrast, the citrate-stabilized AuNPs, used in the preparation of the conjugates and designated as TC-AuNPs, were negatively charged but also stable in the culture media.

The biological studies using the MTT assay showed that CPZ reduced the viability of human lymphocytes (HUT-78 and COLO 720L), as well as human (COLO 679) and murine (B16-F0) melanoma cells in a dose-dependent manner. The cellular response to the CPZ treatment varied between cell types, although the murine melanoma cells were more resistant to its effects than the human cells. Studies conducted on selected AuNPs revealed that their impact on cell viability, when applied at concentrations above 50 mg/L, was relatively minor. Interestingly, changes in the mitochondrial activity of cells after AuNP treatment were lowest for the positively charged cysteamine-stabilized AuNPs with an average size of 15 ± 6 nm. At a given concentration of AuNPs, the cell viability decreased more significantly when treated with CPZ-AuNPs compared to AuNPs alone. It was observed that the CPZ-AuNP conjugates were more toxic to the cells than both negatively and positively charged AuNPs with the same metal core size. The CPZ-AuNP conjugates were found to inhibit mitochondrial activity and disrupt the cell membrane, as evidenced by the measurement of LDH leakage. The observed membrane damage was also a consequence of oxidative stress that was induced during exposure to the conjugates. COLO 720L and COLO 679 cells were found to be more sensitive to membrane damage.

Analyzing the results of the biological studies obtained for the unconjugated CPZ, AuNPs, and CPZ-AuNP conjugates, and taking into account the effect of the gold core size as well as the surface properties of the colloidal systems studied, it was concluded that the conjugation of CPZ to AuNPs increased the toxic effect compared to AuNPs alone. However, when comparing the biological effects caused by CPZ-AuNPs and free CPZ molecules at the same concentration of the drug, it can be stated that conjugation to nanoparticles inhibits the biological activity of this neuroleptic. 

## Figures and Tables

**Table 1:** Selected physicochemical properties of TC-AuNPs and CHSB-AuNPs.

**Abbreviation/Properties [Unit]**	**TC-AuNPs15**	**TC-AuNPs55**	**CHSB-AuNPs15**
Capping agent	trisodium citrate (TC)	trisodium citrate (TC)	cysteamine (CH)
Mass concentration of Au, determined by ICP-OES [mg/L]	185	135	120
Maximum absorption band [nm]	525	530	522
Size/diameter [nm], determined based on TEM micrographs	17 ± 5	55 ± 5	15 ± 6
Polydispersity index (PdI)	0.29	0.09	0.39
Diffusion coefficient [10^−7^ cm^2^s^−1^] *	3.77	1.72	4.29
Hydrodynamic diameter [nm] *	15 ± 4	53 ± 5	11 ± 4
Electrophoretic mobility [(µm cm)(Vs)^−1^] *	−3.21 ± 0.05	−2.82 ± 0.02	1.22 ± 0.05
Zeta potential [mV] *	−56 ± 2	−45 ± 3	27 ± 6

* Determined at pH 7.4 and temperature of 37 °C.

**Table 2:** Selected physicochemical properties of CPZ-AuNP conjugates.

Abbreviation/Properties [Unit]	CPZ-AuNPs15	CPZ-AuNPs55
Concentration of CPZ [mM] (mg/L)	1	1
	(356)	(356)
Mass concentration of Au, determined by ICP-OES [mg/L]	50	50
Maximum absorption bands [nm]	251	250
	314	312
	532	534
Size/diameter [nm], determined based on TEM micrographs	17 ± 5	55 ± 5
Polydispersity index (PdI)	0.29	0.09
Diffusion coefficient [10^−7^ cm^2^s^−1^] *	3.78	1.72
Hydrodynamic diameter [nm] *	15 ± 2	58 ± 8
Electrophoretic mobility [(µm cm)(Vs)^−1^] *	2.84 ± 0.01	2.68 ± 0.02
Zeta potential [mV] *	42 ± 2	32 ± 1

* Determined at pH 7.4 and temperature of 37 °C.

## Data Availability

The original contributions presented in the study are included in the article and Supplementary Materials, further inquiries can be directed to the corresponding author.

## Supplementary Materials

The following supporting information can be downloaded at: https://www.mdpi.com/article/10.3390/ma17194774/s1, Figure S1: Typical TEM micrographs showing (a) TC-AuNPs15, (b) TC-AuNPs55, (c) CHSB-AuNPs15, and (d) size distribution of AuNPs; Figure S2: Deposition kinetics of (a) TC-AuNPs15 and (b) TC-AuNPs55 on PAH-modified Si/SiO_2_ sensors determined using a QCM; Figure S3: Adsorption kinetics of CPZ molecules on (a) TC-AuNPs15 and (b) TC-AuNPs55 monolayers; Figure S4: Effect of CPZ on the viability of (a) lymphocytes and (b) melanoma cells.

## References

[1] López-Muñoz F., Alamo C., Cuenca E., Shen W.W., Clervoy P., Rubio G. (2005). History of the discovery and clinical introduction of chlorpromazine. Ann. Clin. Psychiatry.

[2] Boyd-Kimball D., Gonczy K., Lewis B., Mason T., Siliko N., Wolfe J. (2018). Classics in chemical neuroscience: Chlorpromazine. ACS Chem. Neurosci..

[3] Gnacek P., Piergies N., Niemiec P., Kowalska O., Oćwieja M. (2024). Spectroscopic studies under properties of chlorpromazine conjugated to gold nanoparticles. Spectrochim. Acta A Mol. Biomol. Spectrosc..

[4] Rybakowski J.K. (2023). Application of antipsychotic drugs in mood disorders. Brain Sci..

[5] Munyon W.H., Salo R., Briones D.F. (1987). Cytotoxic effects of neuroleptic drugs. Psychopharmacology.

[6] Lialiaris T., Pantazaki A., Sivridis E., Mourelatos D. (1992). Chlorpromazine-induced damage on nucleic acids: A combined cytogenetic and biochemical study. Mutat. Res./Fundam. Mol. Mech. Mutagen..

[7] Darkin S., McQuillan J., Ralph R.K. (1984). Chlorpromazine: A potential anticancer agent?. Biochem. Biophys. Res. Commun..

[8] Zinetti M., Galli G., Demitri M.T., Fantuzzi G., Minto M., Ghezzi P., Alzani R., Cozzi E., Fratelli M. (1995). Chlorpromazine inhibits tumour necrosis factor synthesis and cytotoxicity in vitro. Immunology.

[9] Lialiaris T.S., Papachristou F., Mourelatos C., Simopoulou M. (2009). Antineoplastic and cytogenetic effects of chlorpromazine on human lymphocytes in vitro and on Ehrlich ascites tumor cells in vivo. Anti-Cancer Drugs.

[10] Kamgar-Dayhoff P., Brelidze T.I. (2021). Multifaceted effect of chlorpromazine in cancer: Implications for cancer treatment. Oncotarget.

[11] Marion P., Attali D., Prot M., Petit A.-C., Blatzer M., Vinckier F., Levillayer L., Chiaravalli J., Perin-Dureau F., Cachia A. (2021). Inhibition of the replication of SARS-CoV-2 in human cells by the FDA-approved drug chlorpromazine. Int. J. Antimicrob. Agents.

[12] Karwaciak I., Karaś K., Sałkowska A., Pastwińska J., Ratajewski M. (2022). Chlorpromazine, a clinically approved drug, inhibits SARS-CoV-2 nucleocapsid-mediated induction of IL-6 in human monocytes. Molecules.

[13] Halayqa M., Domańska U. (2014). PLGA biodegradable nanoparticles containing perphenazine or chlorpromazine hydrochloride: Effect of formulation and release. Int. J. Mol. Sci..

[14] Govender T., Choonara Y.E., Kumar P., Du Toit L.C., Modi G., Naidoo D., Pillay V. (2015). A novel melt-dispersion technique for simplistic preparation of chlorpromazine-loaded polycaprolactone nanocapsules. Polymers.

[15] Baloch J., Sohail M.F., Sarwar H.S., Kiani M.H., Khan G.M., Jahan S., Rafay M., Chaudhry M.T., Yasinzai M., Shahnaz G. (2019). Self-nanoemulsifying drug delivery system (SNEDDS) for improved oral bioavailability of chlorpromazine: In vitro and in vivo evaluation. Medicina.

[16] Panjla A., Kaul G., Akhir A., Saxena D., Joshi S., Modak C., Kumari D., Jain A., Chopra S., Verma S. (2023). Targeting multidrug resistant Staphylococcus aureus with cationic chlorpromazine-peptide conjugates. Chem. Asian J..

[17] Hao X., Liu W., Zhang Y., Kang W., Niu L., Ai L. (2021). A novel and rapid method to detect chlorpromazine hydrochloride in biological sample based on SERS. Chem. Phys. Lett..

[18] Barveen N.R., Wang T.J., Chang Y.H. (2022). A photochemical approach to anchor Au NPs on MXene as a prominent SERS substrate for ultrasensitive detection of chlorpromazine. Microchim. Acta.

[19] Szaniawska A., Mazur K., Kwarta D., Pyrak E., Kudelski A. (2022). How surface-enhanced Raman spectroscopy could contribute to medical diagnoses. Chemosensors.

[20] Chen R., Chen Q., Wang Y., Feng Z., Xu Z., Zhou P., Huang W., Cheng H., Li L., Feng J. (2023). Ultrasensitive SERS substrate for label-free therapeutic drug monitoring of chlorpromazine hydrochloride and aminophylline in human serum. Anal. Bioanal. Chem..

[21] Jebakumari K.E., Murugasenapathi N.K., Palanisamy T. (2023). Engineered two-dimensional nanostructures as SERS substrates for biomolecule sensing: A review. Biosensors.

[22] Turkevich J., Stevenson P.C., Hillier J. (1951). A study of the nucleation and growth processes in the synthesis of colloidal gold. Discuss. Faraday Soc..

[23] Ziegler C., Eychmuller A. (2011). Seeded growth synthesis of uniform gold nanoparticles with diameters of 15–300 nm. J. Phys. Chem. C.

[24] Bubniene U., Oćwieja M., Bugelyte B., Adamczyk Z., Nattich-Rak M., Voronovic J., Ramanaviciene A., Ramanavicius A. (2014). Deposition of gold nanoparticles on mica modified by poly (allylamine hydrochloride) monolayers. Colloids Surf. A Physicochem. Eng. Asp..

[25] Oćwieja M., Maciejewska-Prończuk J., Adamczyk Z., Roman M. (2017). Formation of positively charged gold nanoparticle monolayers on silica sensors. J. Colloid Interface Sci..

[26] Ungor D., Barbasz A., Czyżowska A., Csapó E., Oćwieja M. (2021). Cytotoxicity studies of protein-stabilized fluorescent gold nanoclusters on human lymphocytes. Colloids Surf. B Biointerfaces.

[27] Kubiak K., Adamczyk Z., Ocwieja M. (2015). Kinetics of silver nanoparticle deposition at PAH monolayers: Reference QCM results. Langmuir.

[28] Kula-Maximenko M., Gorczyca A., Pociecha E., Gąstoł A., Maciejewska-Prończuk J., Oćwieja M. (2022). Characterization of selected parameters of Chlorella vulgaris microalgae after short-term exposure to gold nanoparticles with different surface properties. J. Environ. Chem. Eng..

[29] Park J.W., Shumaker-Parry J.S. (2014). Structural study of citrate layers on gold nanoparticles: Role of intermolecular interactions in stabilizing nanoparticles. J. Am. Chem. Soc..

[30] Otto A., Bruckbauer A., Chen Y.X. (2003). On the chloride activation in SERS and single molecule SERS. J. Mol. Struct..

[31] Pisciotta A.V., Westring D.W., Deprey C. (1967). Studies on agranulocytosis. VIII. Inhibition of mitosis in phytohemagglutinin-stimulated lymphocytes by chlorpromazine. J. Lab. Clin. Med..

[32] Ferguson R.M., Schmidtke J.R., Simmons R.L. (1976). Differential effects of chlorpromazine on the in vitro generation and effector function of cytotoxic lymphocytes. J. Exp. Med..

[33] Donner M., Mehrishi J.N. (1980). Binding of chlorpromazine and HLA-A1 antibodies to human lymphocyte membranes. Biochemistry of Schizophrenia and Addiction: In Search of a Common Factor.

[34] Thomas H., Grötsch P., Blank N., Grünke M., Capraru D., Geiler T., Winkler S., Kalden J.R. (2000). Chlorpromazine induces apoptosis in activated human lymphoblasts: A mechanism supporting the induction of drug-induced lupus erythematosus?. Arthritis Rheumatol..

[35] Van Woert M.H., Palmer S.H. (1969). Inhibition of the growth of mouse melanoma by chlorpromazine. Cancer Res..

[36] Parsons P.G., Allen B.J. (1986). Accumulation of chlorpromazine and thiouracil by human melanoma cells in culture. Aust. J. Exp. Biol. Med. Sci..

[37] Gil-Ad I., Shtaif B., Levkovitz Y., Nordenberg J., Taler M., Korov I., Weizman A. (2006). Phenothiazines induce apoptosis in a B16 mouse melanoma cell line and attenuate in vivo melanoma tumor growth. Oncol. Rep..

[38] Korth C., May B.C., Cohen F.E., Prusiner S.B. (2001). Acridine and phenothiazine derivatives as pharmacotherapeutics for prion disease. Proc. Natl. Acad. Sci. USA.

[39] Taniguchi S., Suzuki N., Masuda M., Hisanaga S.I., Iwatsubo T., Goedert M., Hasegawa M. (2005). Inhibition of heparin-induced tau filament formation by phenothiazines, polyphenols, and porphyrins. J. Biol. Chem..

[40] Ielo I., Rando G., Giacobello F., Sfameni S., Castellano A., Galletta M., Drommi D., Rosace G., Plutino M.R. (2021). Synthesis, chemical–physical characterization, and biomedical applications of functional gold nanoparticles: A review. Molecules.

[41] Burlec A.F., Corciova A., Boev M., Batir-Marin D., Mircea C., Cioanca O., Danila G., Danila M., Bucur A.F., Hancianu M. (2023). Current overview of metal nanoparticles’ synthesis, characterization, and biomedical applications, with a focus on silver and gold nanoparticles. Pharmaceuticals.

[42] Vaiman E.E., Novitsky M.A., Nasyrova R.F. (2021). Pharmacogenetics of chlorpromazine and its role in the development of antipsychotic-induced parkinsonism. Pers. Psychiatry Neurol..

[43] Liao Y.H., Chang Y.J., Yoshiike Y., Chang Y.C., Chen Y.R. (2012). Negatively charged gold nanoparticles inhibit Alzheimer’s amyloid-β fibrillization, induce fibril dissociation, and mitigate neurotoxicity. Small.

[44] Monti S., Barcaro G., Sementa L., Carravetta V., Ågren H. (2017). Characterization of the adsorption dynamics of trisodium citrate on gold in water solution. RSC Adv..

[45] Gocke E. (1996). Review of the genotoxic properties of chlorpromazine and related phenothiazines. Mutat. Res./Rev. Genet. Toxicol..

[46] Barbasz A., Oćwieja M., Roman M. (2017). Toxicity of silver nanoparticles towards tumoral human cell lines U-937 and HL-60. Colloids Surf. B Biointerfaces.

