# Supplementary material for: The Adsorption of Chlorpromazine on the Surface of Gold Nanoparticles and Its Effect on the Toxicity to Selected Mammalian Cells
Source: Materials (Basel). 2024 Sep 28;17(19):4774. doi: 10.3390/ma17194774 (PMC11477946; doi:10.3390/ma17194774)
Supplement: Supplementary file 1 [file materials-17-04774-s001.zip › materials-3191719-supplementary.pdf]

Supporting materials

The adsorption of chlorpromazine on the surface of gold nanoparticles and its effect on the toxicity to selected mammalian cells

Magdalena Oćwieja[1*] (magdalena.ocwieja@ikifp.edu.pl), Anna Barbasz[2] (anna.barbasz@up.krakow.pl), Oliwia Kowalska[1] (oliwia.kowalska@ikifp.edu.pl), Julia Maciejewska-Prończuk[3] (julia.maciejewska-pronczuk@pk.edu.pl) , Agata Lada[4] (a_lada@anstar.edu.pl)

[1]*Jerzy Haber Institute of Catalysis and Surface Chemistry, Polish Academy of Sciences, Niezapominajek 8, PL-30239 Krakow, Poland*

[2]*Department of Biochemistry and Biophysics, Institute of Biology and Earth Sciences, University of the National Education Commission, Podchorazych 2, PL-30-084, Krakow, Poland*

[3]*Department of Chemical and Process Engineering, Cracow University of Technology, Warszawska 24, PL-31155 Krakow, Poland*

[4] *Faculty of Mathematics and Natural Sciences, Department of Chemistry, University of Applied Sciences in Tarnow, Mickiewicza 8, 33-100 Tarnow, Poland*

* Corresponding author
Magdalena Oćwieja
Jerzy Haber Institute of Catalysis and Surface Chemistry, Polish Academy of Sciences
Niezapominajek 8, PL-30239 Krakow, Poland
email: magdalena.ocwieja@ikifp.edu.pl
phone: +48126395112

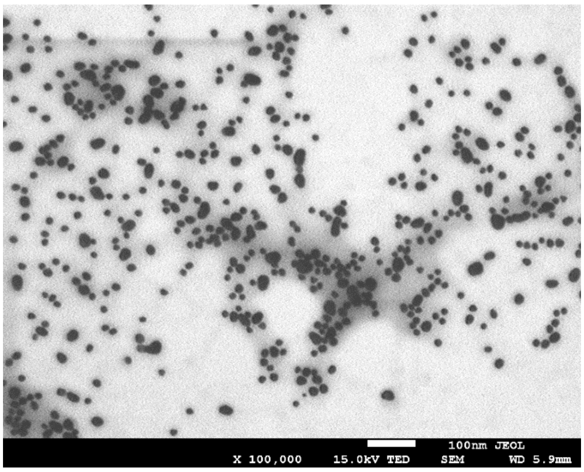

(a)

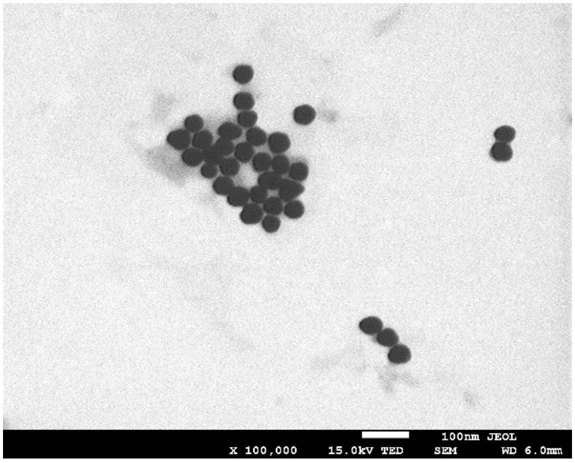

(b)

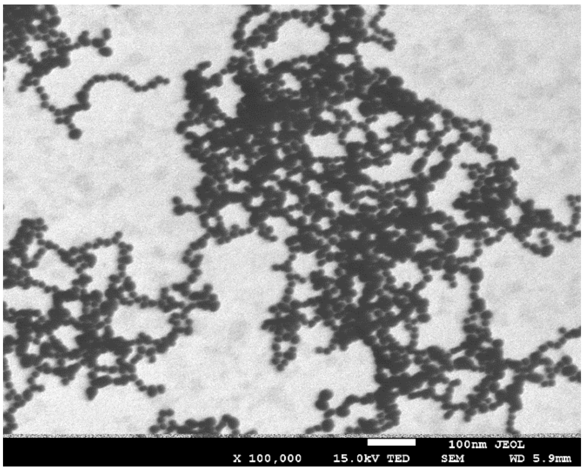

(c)

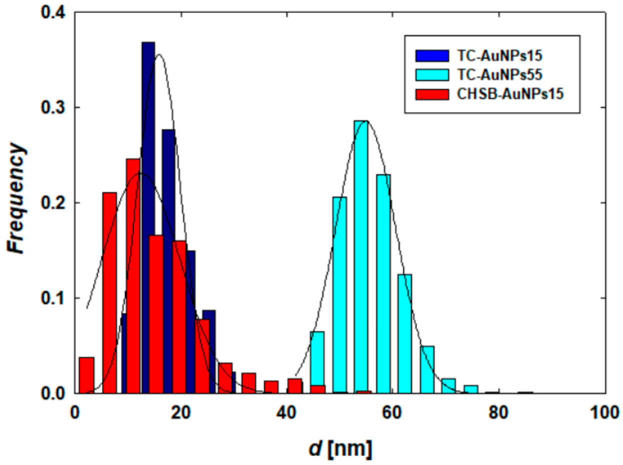

(d)

Figure S1. Typical TEM micrographs showing: **(a)** TC-AuNPs15, **(b)** TC-AuNPs55, **(c)** CHSB-AuNPs15 and **(d)** size distribution of AuNPs. The histograms were prepared by analyzing the diameters of 1000 AuNPs of each type.

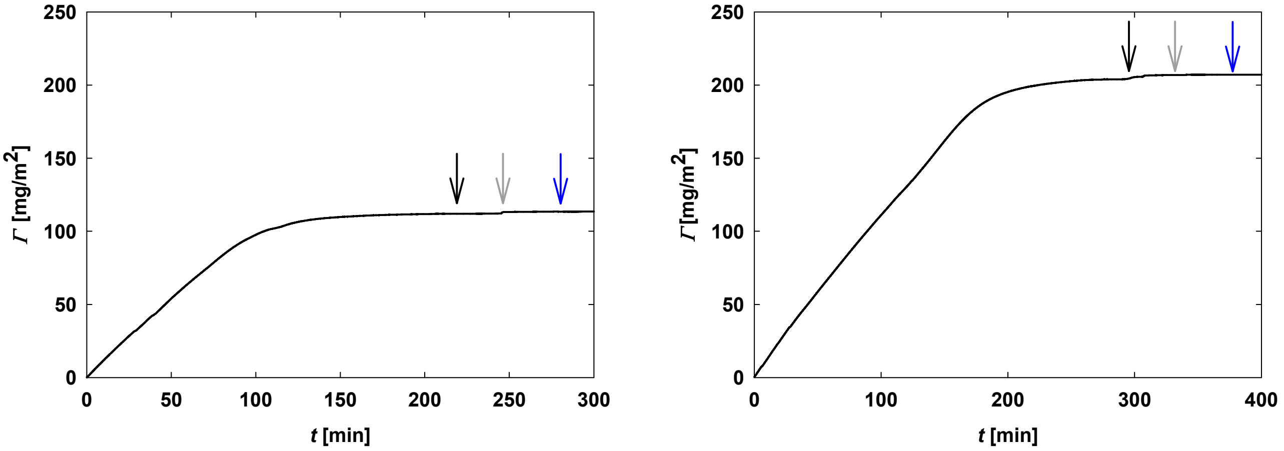

(a) **(b)**

Figure S2. Deposition kinetics of (**a**) TC-AuNPs15 and (**b**) TC-AuNPs55 on PAH-modified Si/SiO_2 sensors determined using a QCM (flow rate 1.33×10^{-3} cm^3/s) for a suspension concentration of **(a)** 30 mg/L and **(b)** 50 mg/L, ionic strength of 10^{-2} M, pH 5.8 and temperature of 25°C. The black arrows indicate the start of surface activation using 10^{-2} M NaCl, while the grey arrows indicate the beginning of CPZ deposition. The blue arrows indicate the onset of the desorption experiments, initiated with a sodium chloride solution of ionic strength 10^{-3} M.

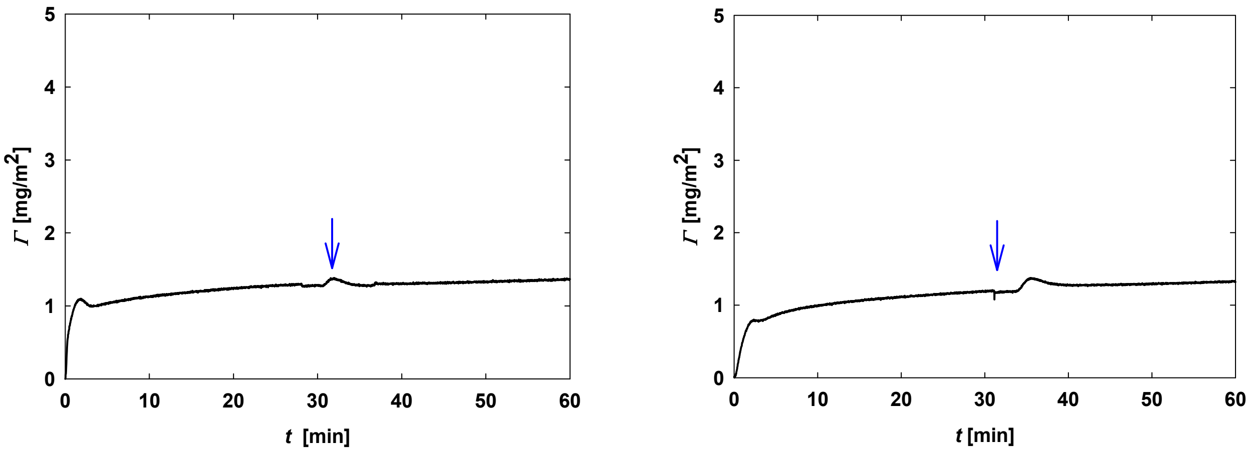

(a) **(b)**

Figure S3. Adsorption kinetics of CPZ molecules on **(a)** TC-AuNPs15 and **(b)** TC-AuNPs55 monolayers with a coverage of 0.32-0.33 (deposited on PAH-modified Si/SiO_2 sensors) were determined using a QCM (flow rate: 1.33×10^{-3} cm^3/s) for a CPZ solution concentration of 10^{-3} M and at a temperature of 25°C. The blue arrows indicate the beginning of the desorption experiments initiated with a sodium chloride solution of ionic strength 10^{-3} M.

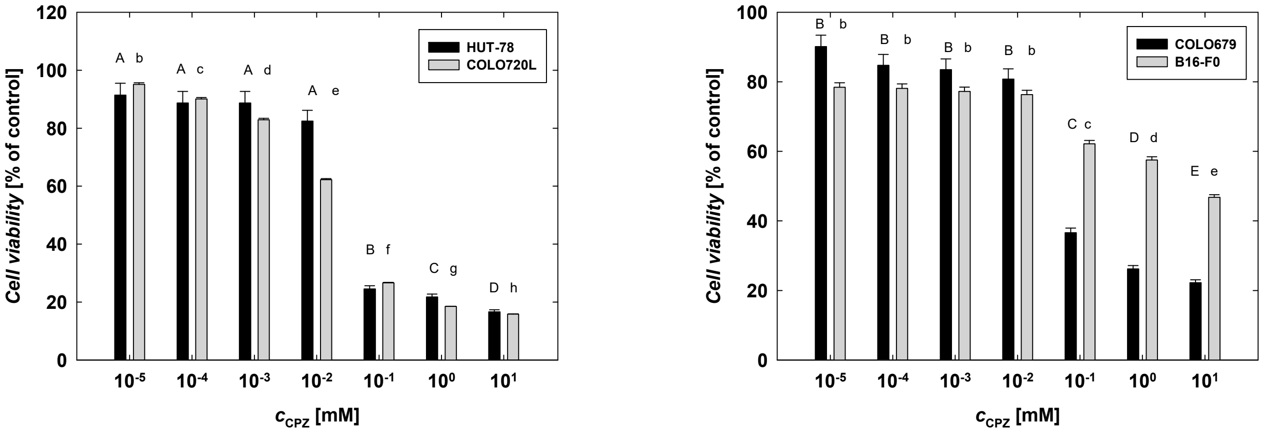

(a) **(b)**

Figure S4. Effect of CPZ on the viability of **(a)** lymphocytes and **(b)** melanoma cells. Cell viability was determined after 24 hours of CPZ treatment using the MTT assay. The letters indicate significant differences ($p < 0.05$) between the results of each treatment.
